# Effect of Tramadol/Acetaminophen on Motivation in Patients with Chronic Low Back Pain

**DOI:** 10.1155/2016/7458534

**Published:** 2016-03-02

**Authors:** Tomoko Tetsunaga, Tomonori Tetsunaga, Masato Tanaka, Keiichiro Nishida, Yoshitaka Takei, Toshifumi Ozaki

**Affiliations:** ^1^Department of Orthopaedic Surgery, Okayama University, 2-5-1 Shikata-cho, Kita-ku, Okayama 700-8558, Japan; ^2^Department of Orthopaedic Surgery, Kurashiki Municipal Hospital, 2-39 Kojima-ekimae, Kurashiki 711-0921, Japan; ^3^Department of Human Morphology, Okayama University, 2-5-1 Shikata-cho, Kita-ku, Okayama 700-8558, Japan

## Abstract

*Background*. The contribution of apathy, frequently recognized in individuals with neurodegenerative diseases, to chronic low back pain (LBP) remains unclear.* Objectives*. To investigate levels of apathy and clinical outcomes in patients with chronic LBP treated with tramadol-acetaminophen.* Methods*. A retrospective case-control study involving 73 patients with chronic LBP (23 male, 50 female; mean age 71 years) treated with tramadol-acetaminophen (*n* = 36) and celecoxib (*n* = 37) was performed. All patients were assessed using the self-reported questionnaires. A mediation model was constructed using a bootstrapping method to evaluate the mediating effects of pain relief after treatment.* Results*. A total of 35 (55.6%) patients met the criteria for apathy. A four-week treatment regimen in the tramadol group conferred significant improvements in the Apathy scale and numerical rating scale but not in the Rolland-Morris Disability Questionnaire, Pain Disability Assessment Scale, or Pain Catastrophizing Scale. The depression component of the Hospital Anxiety and Depression Scale was lower in the tramadol group than in the celecoxib group. The mediation analysis found that the impact of tramadol-acetaminophen on the change in apathy was not mediated by the pain relief.* Conclusions*. Tramadol-acetaminophen was effective at reducing chronic LBP and conferred a prophylactic motivational effect in patients with chronic LBP.

## 1. Introduction

Apathy is broadly defined as a loss of motivation and manifests in behaviors such as diminished motivation, poor persistence, lack of interest, indifference, low social engagement, blunted emotional response, and lack of insight [1]. Apathy is a common feature of neurodegenerative disorders such as Alzheimer's disease [2] and Parkinson's disease [3]; however, there is no consensus regarding the diagnostic criteria for apathy, and the presence of confounding factors, such as depression, makes it difficult to study.

Chronic low back pain (LBP) patients have shown higher ratings of pain intensity and more comorbidities such as depression, panic/anxiety, and sleep disorders [4]. Psychological factors, occupational disability, and somatization disorder have the potential to result in prolonged LBP [5]. Apathy is also increasingly recognized as a common behavioral syndrome [6], and it is associated with negative effects including cognitive decline [7] and decreased functioning [8]. However, the prevalence of apathy in chronic pain has not been studied. Apathy and depressive mood are the two core elements of depression [9]. Apathy is more frequently associated with functional abilities and interacts more with the recovery process than depression [10]. Therefore, apathy disturbs not only the treatment of depression, but also the restoration of physical function [11].

We previously reported antidepressant-like effects of tramadol-acetaminophen in chronic LBP patients with depression [12]. The *μ*-opioid agonist activity of tramadol conceivably plays a role in mood improvement [13, 14]. Low noradrenaline levels have been significantly associated with not only depression, but also apathy [15]. The motivational effects of tramadol-acetaminophen in patients with chronic LBP remain unclear. The purpose of the present study was to investigate the frequency of apathy in chronic LBP and the therapeutic efficacy of tramadol-acetaminophen as a treatment for pain and apathy in patients with chronic LBP.

## 2. Methods

### 2.1. Patients

Performed at the authors' institution, the present four-week study compared the efficacies of tramadol-acetaminophen and celecoxib in the treatment of patients with chronic LBP. A total of 73 patients (23 men, 50 women) with chronic LBP were included. Inclusion criteria were individuals whose pain had persisted for >3 months and who agreed to answer the questionnaire. Exclusion criteria were patients with dementia, delirium, or other conditions that made it difficult to complete a self-reported written questionnaire. In addition, patients with severe chronic disease (e.g., cardiovascular disease, renal failure, or other disqualifying conditions) that interfered with treatment were also excluded. This was a retrospective case-control study, in which the case group consisted of patients treated with tramadol-acetaminophen tablets (TRAMCET combination tablets; Janssen Pharmaceutical K.K., Tokyo, Japan, tramadol group (*n* = 36)), and the control group consisted of patients treated with celecoxib (Celecox; Astellas Pharma Inc., Tokyo, Japan, celecoxib group (*n* = 37)) for four weeks. Patients in the tramadol group took two tramadol-acetaminophen tablets per day. The doses of tramadol-acetaminophen were titrated at the one-week visit up to four tablets per day unless side effects prevented the dose from being titrated further. Patients in the celecoxib group took two celecoxib tablets per day (200 mg/day) for four weeks. No other analgesics or anti-inflammatory medications were administered. Visits were scheduled for days 7, 14, and 28. All patients included in the present study provided written, informed consent. The study was approved by the Kurashiki Municipal Hospital Research Ethics Committee and was conducted according to the Declaration of Helsinki.

### 2.2. Outcome Measures

The primary outcome measure was apathy after four weeks of treatment. Secondary outcome measures included pain, physical disability, anxiety, depression, and pain catastrophizing assessment after four weeks of treatment. Assessments were conducted at baseline and after intervention (4 weeks).

### 2.3. Assessment of Apathy

Apathy was assessed using the Japanese translation of the 14-item Starkstein Apathy Scale [16]. This scale is a modified version of the Apathy Evaluation Scale (AES) [17], which is the most widely used, well-validated, empirically reliable scale to assess general apathy. Each item is rated using a four-point scale (0 = not at all true/characteristic to 3 = very much true/characteristic). The total scores range from 0 to 42, with higher scores indicating more severe apathy. The original cut-off score was 14 points; however, 16 points were used as the cut-off, in accordance with Okada et al. [16].

### 2.4. Pain Assessment

The numerical rating scale (NRS) for pain self-assessment is a widely used, valid, and reliable tool to measure chronic pain intensity [18]. The NRS score ranges from 0 to 10, with 0 representing no pain and 10 representing the worst pain imaginable. The NRS score was obtained at baseline and after four weeks of treatment.

### 2.5. Physical Disability Assessment

Self-reported disability due to LBP was assessed using the Roland-Morris Disability Questionnaire (RDQ). The RDQ is widely used to assess physical disability associated with back pain and has been shown to be valid, reliable, and responsive to treatment [19]. The RDQ is scored on a scale of 0 to 24, with higher scores indicating greater physical disability. The RDQ score was obtained at baseline and after four weeks of treatment. The Pain Disability Assessment Scale (PDAS) contains items that assess the negative effects of pain on broad-spectrum pain interference domains [20]. The PDAS consists of 20 items scored using a four-point Likert scale from 0 to 3, with scores ranging from 0 to 60. This scale is useful when clinicians require a multidimensional measure of the effects of pain on a patient's life. The PDAS score was obtained at baseline and after four weeks of treatment.

### 2.6. Anxiety and Depression Assessment

Anxiety and depression were assessed using the Hospital Anxiety and Depression Scale (HADS) [21]. The HADS is very useful in the assessment of anxiety and depression in patients with physical illness. It is a 14-item scale, with seven items assessing anxiety and seven assessing depression. Each item is scored from 0 to 3 using a Likert scale. Overall scores of anxiety or depression can assume values between 0 and 21, with higher scores indicating greater severity of symptoms. The HADS score was obtained at baseline and after four weeks of treatment.

### 2.7. Pain Catastrophizing Assessment

Self-reported pain catastrophizing due to LBP was assessed using the Pain Catastrophizing Scale (PCS) [22]. The PCS is a broad measure of pain catastrophizing and consists of 13 items scored using five-point Likert scales ranging from 0 (never) to 4 (always) points. The maximum score for the PCS is 52, with higher scores indicating greater pain catastrophizing levels. A score >24 indicates a high level of catastrophizing. The items are divided into three subscales: rumination, helplessness, and magnification. Rumination (items 8 to 11) “refers to the fact that the patient cannot get the idea of pain out of his/her head and cannot stop thinking about the pain”; helplessness (items 1 to 5 and 12) “refers to the estimation that the person has of not being able to do anything to influence the pain”; and magnification (items 6, 7, and 13) “refers to the exaggeration of the threatening properties of the painful stimulus.” High internal reliability has been reported in patients with chronic pain, with adequate validity and test-retest reliability [23]. The PCS score was obtained at baseline and after four weeks of treatment.

### 2.8. Statistical Analysis

Normally distributed variables were compared using Student's *t*-test. The chi-squared test was used to compare differences in the sex ratio. Differences in data between groups after treatment were tested by repeated measures analysis of variance (ANOVA) with Tukey's post hoc test. Cohen's *d* was calculated to evaluate effect sizes. Differences with *P* < 0.05 were considered significant. Statistical analysis was conducted using SPSS version 18 for Windows (IBM Corporation, Tokyo, Japan). A pilot study to calculate the required sample size per group was performed. This pilot study included 10 patients in each group. The results of the pilot study indicated that the Apathy Scale decreased from 16 points to 14.3 points after treatment, with a standard deviation of 5.0. The Apathy Scale was decreased from 15.8 points to 12.5 points in the tramadol group and from 16.2 points to 16 points in the celecoxib group after treatment. A power analysis (using means of 12.5 points [tramadol group] and 16 points [celecoxib group] with a standard deviation of 5.0) estimated that 33 patients would be needed in each group to provide a 95% chance of detecting such a reduction at the 0.05 level of significance. Mediation analysis was used to evaluate the direct effects of tramadol-acetaminophen on the change in apathy and the effects mediated by the pain relief, along with their standardizing coefficient (*b*), using bootstrapping (with 2000 replications) and 95% confidence intervals (CIs). An indirect effect (tramadol-acetaminophen affects the pain relief, which in turn affects the change in apathy) was considered significant if the CI did not contain zero.

## 3. Results

### 3.1. Patient Demographics

The mean duration from symptom onset to consultation was 50 months (range 3 to 240 months); the mean age at the time of examination was 71 years (range 36 to 95 years) (Table 1). In the present series, 32 patients had osteoarthritis, 18 had lumbar canal stenosis, 12 had lumbar disc herniation, 6 had degenerative spondylolisthesis, and 5 were in postspinal surgery phase. There were 12 male and 24 female patients, with a mean age of 71 years (range 36 to 95 years) in the tramadol group. There were 11 male and 26 female patients, with a mean age of 70 years (range 45 to 91 years) in the celecoxib group. The mean pain duration was 49.6 months (range 3 to 240 months) in the tramadol group and 50.7 months (range 3 to 180 months) in the celecoxib group. No significant differences between the two groups were observed with regard to age (*P* = 0.37) and pain duration (*P* = 0.47). In the present study, 35 (55.6%) patients met the criteria of apathy based on the Starkstein Apathy Scale. There were no significant differences between the two groups in terms of mean Apathy Scale scores (*P* = 0.47), NRS (*P* = 0.08), RDQ (*P* = 0.92), PDAS (*P* = 0.56), HADS (anxiety, *P* = 0.63; depression, *P* = 0.89), and PCS (*P* = 0.99) scores.

### 3.2. Treatment Effectiveness with Tramadol-Acetaminophen or Celecoxib

The Apathy Scale score was significantly lower in the tramadol group than in the celecoxib group after treatment (*P* < 0.05). The NRS was significantly lower in the tramadol group than in the celecoxib group after treatment (*P* < 0.05), suggesting enhancement of internal pain control by tramadol-acetaminophen (Table 2). There were no significant differences between the treatment groups in RDQ and PDAS scores after four weeks of treatment (*P* = 0.08, 0.77, resp.). No significant difference was found between the treatment groups in the mean HADS anxiety scores (*P* = 0.48) after four weeks of treatment, while a significant difference was found in the mean HADS depression score (*P* < 0.05), suggesting that tramadol-acetaminophen treatment has an antidepressant effect. There was no significant difference between the groups in the PCS scores after four weeks of treatment (*P* = 0.35).

### 3.3. Mediation Analysis

**Figure 1:**
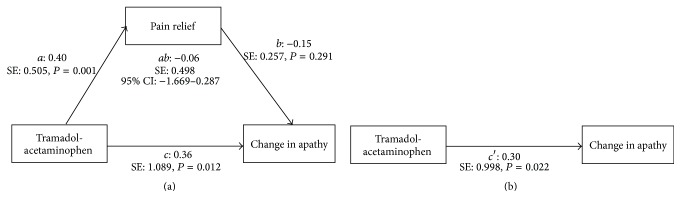
Standardized coefficients mediation models showing the indirect (a) and direct (b) effects of tramadol-acetaminophen on change in apathy. *a*, *b*, *c*, and *c*′ indicate standardized coefficients. SE: standard error; 95% CI: 95% confidence interval.

A mediation model to test whether the effect of the tramadol-acetaminophen on the change in apathy is mediated by the pain relief was constructed (Figure 1). The direct effect of tramadol-acetaminophen on the change in apathy remained significant (*b*: 0.30, *P* < 0.05). On the other hand, the bootstrapping method showed that the 95% CI contained zero (95% CI: −1.669–0.287), indicating that the effect of tramadol-acetaminophen on the change in apathy was not mediated by the pain relief. The results of mediation analysis showed that tramadol-acetaminophen was associated with the change in apathy directly, not through pain relief.

### 3.4. Adverse Events

Fourteen (38.9%) patients reported adverse events during tramadol-acetaminophen treatment, and nine (24.3%) patients reported adverse events during celecoxib treatment. The difference in the overall incidence of adverse events between the treatment groups was not significant (*P* = 0.18). The adverse events related to the study medication for both groups are summarized in Table 3. In the present study, no patients in either group withdrew because of adverse events.

## 4. Discussion

In this study, the frequency of apathy was investigated in chronic LBP patients. According to the Apathy Scale, apathy was present in 55.6% of patients with chronic LBP. This result was comparable to that in patients with Alzheimer disease (60%) [2] and Parkinson disease (52.2%) [3]. In the present study, the combination of tramadol and acetaminophen showed efficacy not only for the reduction of pain intensity, but also for motivation, with tolerable side effects during the course of treatment. Although nausea (22.2%), somnolence (8.3%), and constipation (5.6%) were noted as side effects of tramadol-acetaminophen, all episodes of somnolence and nausea were transient, and no patients had to discontinue treatment.

Because most pain conditions involve several different pathways, analgesic therapy with a single agent may be inadequate to relieve chronic pain. Combination analgesics with two or more agents may have synergistic analgesic effects and may provide more effective pain relief for a broader spectrum of pain [24]. Dosages of tramadol and acetaminophen that are ineffective when administered separately provide adequate pain relief when combined through the actions of different pathways and reduce the incidence of adverse events [24]. Tramadol is popular because of its low potential for addiction and its quick-acting properties compared with other opioid analgesics [25]. Furthermore, acetaminophen is a shorter- and faster-acting analgesic than tramadol [26]. In the present study, tramadol-acetaminophen provided good pain relief in patients with chronic LBP.

Pincus et al. [27] reported that there was robust evidence supporting the role of negative mood (distress or depression) in the transition to chronic pain status. Psychological factors are important contributors to the transition to chronic LBP [27]. In addition to long-term symptoms, the combination of chronic pain, anxiety/depression, and sleep disorders, also referred to as the pain triad, causes functional impairment [28]. Thus, in addition to a biological approach, a psychological approach is necessary in these cases. In the treatment process for depression, physical symptoms such as insomnia and depressed mood often recover at a relatively early stage, while apathy follows a protracted course. Apathy is more frequently associated with functional abilities and interacts more with the recovery process than depression [10]. Therefore, apathy disturbs not only the treatment of depression, but also restoration of physical function [11]. There would be different neuroanatomical mechanisms for depression and apathy, because the severity of depression is associated with left frontal lobe damage, while that of apathy is related to damage to the bilateral basal ganglia [29]. Therefore, the prevalence of apathy was investigated in chronic LBP patients in this study. In the present study, the apathy score decreased significantly in the tramadol group after four weeks of treatment. However, apathy appears to provoke a symptom spiral, which leads to the question of whether pain increases apathy or apathy increases pain by inactivity and reduced mobilization [30]. Regularly scheduled administration of acetaminophen can lead to significant behavior change by relief of chronic pain in nursing home residents with moderate-to-severe dementia, particularly regarding activity levels and social engagement [31]. Although both apathy and pain were improved after treatment in the tramadol group, the results of mediation analysis showed that improvement of apathy after treatment was not mediated through the pain relief. Tramadol is a centrally acting analgesic with weak *μ*-opioid agonist effects and weak inhibition of serotonin and norepinephrine reuptake [32]. This *μ*-opioid agonist activity plays a role in mood improvement [13, 14]. We consider that the motivational effect observed in the present study might be explained by direct effects of tramadol/acetaminophen, not indirect effects attributed to the pain relief.

Patients with chronic pain frequently complain of depression and sleep problems. Depressive conditions may lead to reductions in the pain threshold [33]; therefore, patients experience continuing pain with prolonged therapy. Although many patients treated in the present study had depressive conditions before treatment, the HADS depression score was significantly decreased in the tramadol group after four weeks of treatment. This result suggests that tramadol-acetaminophen may have antidepressant-like activity. However, the NRS score after treatment was also significantly lower in the tramadol group than in the celecoxib group. The significant pain relief observed in the tramadol group may have an effect on the secondary reduction of the depression score. There are several reports in the literature that are consistent with the present results. Tramadol had antidepressant-like effects in animal models of depression, mediated by interaction with the noradrenergic system [34–36], and was comparable to that of fluoxetine, a selective serotonin reuptake inhibitor [37]. Reeves and Cox [38] reported that a patient with chronic LBP who underwent a laminectomy for a lumbar disc herniation developed significant depression following cessation of tramadol after several years of therapy. These clinical results may indicate that tramadol has an antidepressant effect in patients with chronic pain and support the results of the current study.

The triad of pain, mentioned above, causes functional impairment in many areas of life [28]. The mean HADS depression score was significantly lower in the tramadol group than in the celecoxib group. These results may be explained by the antidepressant-like activity of tramadol-acetaminophen [35, 36]. The fear-avoidance model is a cognitive behavioral account that explains the transition to intractable chronic pain [39]. In this model, both anxiety and catastrophizing, which is associated with subsequent intolerance to activity [40], play an important role. In this short-term study, there were no significant differences in the anxiety, pain disability, and pain catastrophizing scores between the two groups. Considering the fear-avoidance model, reduction of pain and depression may impact other factors such as anxiety, pain disability, and pain catastrophizing positively. However, significant improvement of these factors was not seen in another study, despite providing a longer treatment period [12]. A multidisciplinary approach has been shown to be effective for the treatment of intractable pain. Formal liaisons between general practitioners and specialists have been demonstrated to improve functional outcomes in chronically mentally ill patients [41]. A liaison clinic for patients with intractable chronic pain was able to improve patient catastrophizing and anxiety [42]. The results of this study suggest that the multidisciplinary intervention may be effective in treating anxiety, pain disability, and pain catastrophizing.

The current study had some limitations. First, the dose of tramadol-acetaminophen was changed at the one-week visit from two tablets per day to four tablets per day unless side effects were experienced or adverse events occurred. In contrast, patients in the celecoxib group remained on two celecoxib tablets per day (200 mg/day) for the entire duration of the study. Thus, the incidence of adverse drug reactions may be attributed not to the drugs themselves, but rather to the difference in dose. Second, the present study included patients with LBP caused by various degenerative lumbar diseases consisting of a high proportion of lumbar canal stenosis. To evaluate the outcomes of tramadol-acetaminophen more precisely, it would be desirable to study patients with specific lumbar diseases. Third, the Japanese translation of 14-item Starkstein Apathy Scale was used in this study. The AES, Neuropsychiatric Inventory (NPI) [43], and its variation (14-item Starkstein Apathy Scale) are validated for broad application across dementia and cognitively impaired populations [2]. Broad apathy measures such as AES and NPI may not be able to sufficiently detect subtle, disease-specific variations in the presentation of apathy in certain populations (chronic pain patients). Because a scale evaluating apathy in chronic pain has not yet been developed, the Apathy Scale reported by Okada et al. [16] was used in this study. However, because this scale was developed to evaluate poststroke apathy, it has not been validated for chronic pain. Since there are no gold standard apathy assessment tools because of the vague definition of apathy, studies in this field may have been delayed. Finally, tramadol-acetaminophen was administered to patients with chronic pain in the present study. To strictly evaluate the motivational effects of tramadol-acetaminophen, it should be administered to apathetic patients without chronic pain. Despite these limitations, however, the NRS score and the HADS depression and Apathy Scale scores were significantly decreased in the tramadol group after four weeks of treatment (*P* < 0.05). The results of the current study suggest that tramadol-acetaminophen in tablet form may represent an attractive alternative treatment option for chronic LBP patients with depression or apathy.

## Figures and Tables

**Table 1:** Patients' demographics.

Variable	Total	Tramadol	Celecoxib	*P*
	(*n* = 73)	(*n* = 36)	(*n* = 37)	
Age (years)	70.9 ± 12.3	71 ± 11.5	70.4 ± 13	0.37
Male/female	23/50	12/24	11/26	0.74
Pain duration (months)	50 ± 54.5	49.6 ± 58.3	50.7 ± 52.4	0.47
Apathy Scale (points)	15.8 ± 6.6	16.2 ± 5.3	15.5 ± 6.1	0.47
NRS	7.1 ± 1.9	7.5 ± 1.4	6.9 ± 2.1	0.08
RDQ (points)	12.2 ± 5.9	12.3 ± 5.7	12.1 ± 5.4	0.92
PDAS (points)	29.8 ± 15.3	31.7 ± 15.5	28.6 ± 15.3	0.56
HADS anxiety (points)	7.8 ± 4.6	8.1 ± 4.6	7.6 ± 4.1	0.63
HADS depression (points)	7.2 ± 4.0	7.2 ± 3.8	7.1 ± 3.8	0.89
PCS (points)	35.1 ± 12.9	35.1 ± 12	35.1 ± 12.1	0.99

NRS: numerical rating scale; RDQ: Rolland-Morris Disability Questionnaire; PDAS: Pain Disability Assessment Scale; HADS: Hospital Anxiety and Depression Scale; PCS: Pain Catastrophizing Scale; data presented as mean ± SD unless otherwise indicated.

**Table 2:** Apathy, NRS, RDQ, PDAS, HADS, and PCS scores after four-week treatment in the tramadol and celecoxib groups.

Variable	Tramadol (*n* = 36)	Celecoxib (*n* = 37)	*P*	Cohen's *d*	95% CI	
					Lower	Upper
Apathy Scale (points)	12.8 ± 5.7	15.9 ± 6.2	<0.05	0.59	0.31	6.6
NRS	3.7 ± 1.8	4.5 ± 1.9	<0.05	0.64	0.23	2.18
RDQ (points)	8.5 ± 5.3	11.2 ± 6.3	0.08	0.46	−0.41	5.75
PDAS (points)	25.9 ± 16	26.3 ± 14	0.77	0.08	−6.46	8.72
HADS anxiety (points)	5.8 ± 4.1	6.6 ± 4.3	0.48	0.19	−1.41	3.01
HADS depression (points)	4.9 ± 3.1	6.9 ± 4.1	<0.05	0.55	0.07	3.98
PCS (points)	28 ± 13.1	29.9 ± 12.5	0.35	0.24	−3.54	9.6

NRS: numerical rating scale; RDQ: Rolland-Morris Disability Questionnaire; PDAS: Pain Disability Assessment Scale; HADS: Hospital Anxiety and Depression Scale; PCS: Pain Catastrophizing Scale; data presented as mean ± SD unless otherwise indicated.

**Table 3:** Incidence of adverse events related to study medication.

Event	Incidence, *n* (%)			
	Tramadol (*n* = 36)		Celecoxib (*n* = 37)	
Nausea	8	(22.2)	1	(2.7)
Somnolence	3	(8.3)	1	(2.7)
Gastrointestinal disorder	1	(2.8)	3	(8.1)
Constipation	2	(5.6)	1	(2.7)
Dizziness	2	(5.6)	0	(0)
Urtication	1	(2.8)	2	(5.4)
Edema	1	(2.8)	1	(2.7)
